# Peri-implant distal clavicle fracture: Case report (overlaying plate fixation: Solution for peri-implant clavicle fractures)

**DOI:** 10.1016/j.ijscr.2021.106411

**Published:** 2021-09-15

**Authors:** Guilherme Vieira Lima, Nataniel Sousa Santos Filho, Cézar Augusto Pimentel Furlan, Joel Murachovsky, Vitor La Banca, Roberto Yukio Ikemoto

**Affiliations:** a Department of Shoulder and Elbow Surgery, Faculdade de Medicina do ABC, Santo André, Brazil; b Medical Doctor-Shoulder and Elbow Surgeon, Brazil; c Shoulder and Elbow Surgeon Fellowship, Brazil

**Keywords:** Clavicle, Fracture, Bone plate, Osteosyntheses, Complications, Case report

## Abstract

**Introduction and importance:**

Surgical treatment for clavicle injuries is indicated for displaced and shortened fractures. Osteosyntheses with plate fixation may present with complications in 6.3% to 8.5% of patients. Peri-implant clavicle fractures (PIF) are rare, and we have not found any previous cases in our literature search.

**Case presentation:**

A 25-year-old male with previously (six years earlier) surgically treated clavicle fracture presented with a peri-implant clavicle fracture requiring surgical treatment. The management involved overlaying an implant to fix the lateral clavicle fracture without removing the previous plate. Complete bone healing was observed without any further complication.

**Clinical discussion:**

Despite the low rate of implant failure in clavicle fractures, this complication occurs mainly in elderly patients with poor bone quality. No PIF have been described in the literature prior to this. This case report demonstrates a young patient with good bone quality and previous fracture fixation presenting with PIF which has now shown complete bone healing.

**Conclusion:**

In this case, overlying an additional plate on the lateral clavicle portion without removing the previous plate increased the stability of the fracture. It demonstrates the value of overlaying plate osteosyntheses for patients with clavicle PIF.

## Highlights

• Peri-implant clavicle fracture is a rare situation.
• The management of peri-implant fracture is challenging.
• Overlaying plate fixation can be the solution for peri-implant clavicle fractures.

## 1 Introduction

Clavicle fractures are common traumatic lesions; comprising 5–10% of all fractures and 44% of shoulder fractures [1]. Almost 80% of clavicle fractures involve the middle third, and most are treated non-surgically [2].

However, some variations require surgical treatment such as an open fracture or imminent exposure, neurovascular involvement, shortening greater than two centimeters, and comminution with rotation of the fragments [2], [3], [4]. Clavicle osteosynthesis can be done with intramedullary implants such as nails and screws or cortical implants such as plates and external fixation. Despite high success rates, plate fixation has a reported complication rate of 6.3–8.5% [5], [6], [7]. The most frequent are implant loosening, infection, implant discomfort, non-union, and refracture after plate removal [4]. Broken plate is uncommon and occurs more often when reconstruction plates are used [8]. Peri-implant fractures (PIF) of the clavicle are rare, and no reports were found in the researched literature. This case report presents an unusual occurrence in a patient six years postoperatively from a mid-third clavicle fracture who, after another trauma, presented a PIF in the third distal clavicle with displacement that required surgical treatment.

The SCARE 2020 [9] protocol was used as a guideline for this case report.

## 2 Presentation of case

A 25 year old male, white, right-handed motorcycle deliveryman fell off his bike, presenting pain and deformity in his right shoulder and unable to lift his arm. He had previously (six years earlier) surgically treated clavicle fracture due to a skateboard fall. The patient has no relevant history of medication, disease, family illnesses or psychosocial disturb.

**Fig. 1:**
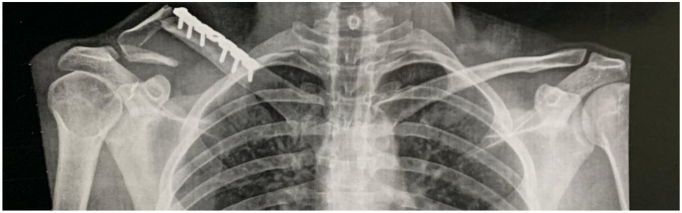
Initial radiographic exam with PIF of the distal third of the clavicle.

Radiographic standard exams were taken at the emergency department showing a clavicle PIF (Fig. 1). The patient presented with no neurologic deficits, other than longstanding localised skin numbness that was present following initial injury six years previously.

**Fig. 2:**
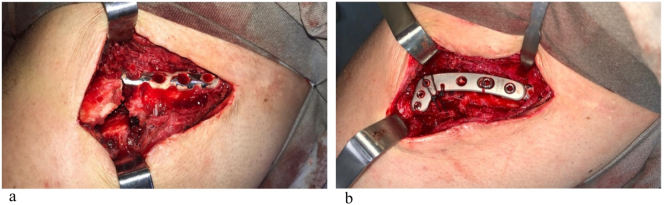
a) Intraoperative image of the fracture with previous plate. b) Final fixation with the overlaying lateral plate and non-absorbable suture.

The patient was referred for surgical treatment with open reduction and internal fixation (ORIF). The proposed surgical management included removing the previous plate and using a new longer plate, including the distal third of the clavicle. The surgery was planned and performed by senior orthopaedic shoulder surgeon. During the procedure, the surgeon chose to perform the fixation without removing the previous plate. The lateral screws were removed from the previous plate, and an overlaying plate was used to fix the fracture. An Ethibond EXCEL® suture was performed for better fixation of the lateral fragment (Fig. 2a and b).

The arm was placed on a sling and allowed a passive range of motion of the hand, wrist, and elbow. Analgesic and anti-inflammatory medication was prescribed for pain control and antibiotics for infectious prophylaxis. After surgery the patient reported few painful events, which were controlled with analgesic (Tylenol®). He also showed good tolerance to the sling without any further complaining. We used the UCLA and CONSTANT scores for postoperative evaluation. The skin sutures were removed after two weeks while maintaining the sling and allowed passive range of motion of the shoulder and active movement of the hand, wrist, and elbow. At this point, UCLA scored 26, and CONSTANT scored 47.

After 30 days of surgery, the sling was removed and the patient presented a wide shoulder passive range of shoulder motion with no complains. UCLA scored 26, and CONSTANT scored 73. Follow-up radiographs showed stable fixation with signs of bone healing.

**Fig. 3:**
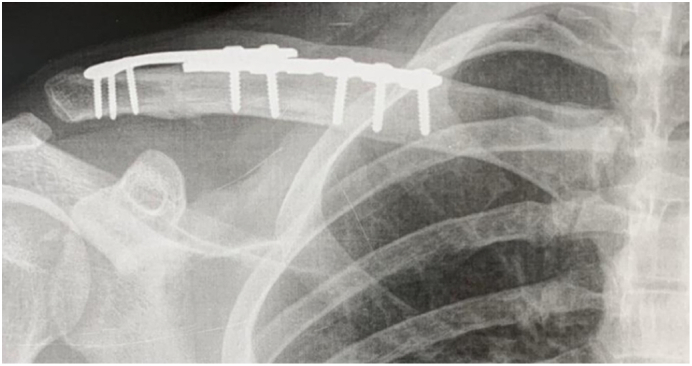
Radiograph at 3-month follow-up showing complete bone healing.

**Fig. 4:**
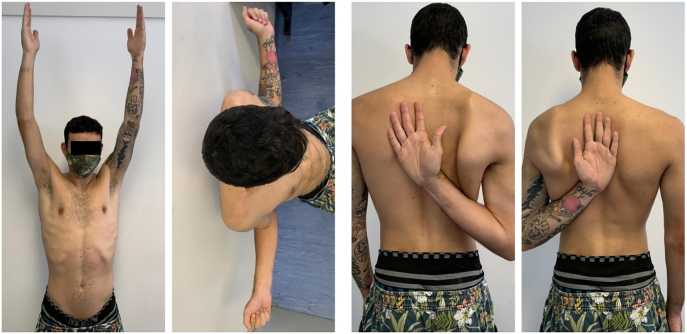
Clinical results at the one-year follow-up. Complete recovery of the elevation, external and internal rotation of the shoulder.

At 90 days postoperatively, the radiographs showed complete bone healing (Fig. 3) with no complaints from the patient. At this point, he was able to return to work. At the sixth month follow-up, he was allowed to return to all sports activities, including skateboard and the UCLA scored 34 and Constant scored 84. One year after surgery, the patient presented the same radiographic and clinical patterns with complete satisfaction, overestimating his expectations. The range of motion was complete (Fig. 4), UCLA scored 35 and Constant scored 94.

The research ethics committee approved this study according to the Helsing protocol.

## 3 Discussion

The treatment of clavicle fractures remains controversial. Non-surgical treatment is indicated for non-displaced fractures, whereas ORIF with plate and screws are frequently used for displaced fracture [2], [4].

Despite the low rate of implant failure in clavicle fractures, this complication occurs mainly in elderly patients with poor bone quality. Meeuwis et al. [3] published in 2007 a retrospective cohort study with 259 patients who underwent surgery for middle third clavicle fractures with reconstruction plates (50 patients = 19%) and clavicle locking plates (209 patients = 81%). Eighteen patients (6,9%) presented failure being 5 for broken plate (4 reconstruction plates and one locking plate), and 13 presented screw loosening. No PIF were described in this review. The current case report demonstrates a young patient with good bone quality and previous fracture fixation with signs of complete bone healing. In this case, the initial plate probably weakened the PIF area and predisposed to further injury.

Furthermore, as Batash et al. [10] reported, the biomechanical behavior of the clavicle is different from a long vertical bone. In the long vertical bone, gravity is applied by compression forces. In the clavicle, gravity is perpendicular to the bone due to its horizontal position. Such behavior may favor fractures in its areas of fragility.

During pre-operative planning, consideration was given to removing the previous plate and using a new longer plate covering the fracture area. However, even with the longest plate, it would produce a new fragile area in the middle third of the clavicle above the previous fracture, leaving the bone at risk again. During surgery, the lateral screws were removed, and a new locking plate was placed over the previous one using the same previous lateral roles. Then the fracture was reduced and stabilized without creating a new fragile area in the clavicle. This decision also contributed to a smaller surgical incision and less soft tissue damage.

Therefore, the intra-operative decision favored the objective to aggregate the maximum stability on the most extended portion of the clavicle without leading to new failure risks.

Tsai et al. [11] showed that up to 20% of patients require repeated intervention due to implant discomfort at 12 months follow-up. In the presented case, the plates did not cause any problems such as implant loss, pain, decreased range of motion, loss of strength or manly skin irritation or discomfort.

The management of PIF is challenging. From this case we observed again how import surgical planning is, particularly implant template planning and biomechanical knowledge.

## 4 Conclusion

A PIF on the clavicle is a rare event. During the surgical treatment for a dislocated fracture, it is mandatory to create stability without leading to new risks of failure. In the case reported it was decided to improve stability with an additional overlying plate on the lateral clavicle portion without removing the previous plate. Complete bone healing was observed without any further complication. Therefore, the authors believe that overlaying plate osteosyntheses can be an option for clavicle PIF.

## Source of funding

None.

## Ethical approval

Approved by: SECRETARIAT OF STATE OF HEALTH (Proponent Institution). SECRETARIA DE ESTADO DA SAUDE – São Paulo, SP BRASIL. E-mail: cep.hipiranga@gmail.com Registration - CAAE: 39427720.3.0000.5488 Número do Parecer: 4.379.

## Consent

Written informed consent was obtained from the patient for publication of this case report and accompanying images. A copy of the written consent is available for review by the Editor-in-Chief of this journal on request.

## CRediT authorship contribution statement

**Guilherme Vieira Lima**: Conceptualization, Methodology, Writing - Original Draf, Writing - Review & Editing.

**Natanael Sousa Santos Filho**: Conceptualization, Writing - Original Draft, Investigation.

**Cézar Augusto Pimentel Furlan**: Visualization, Investigation.

**Vitor LaBanca**: Formal analysis, Data Curation.

**Joel Murachovsky**: Project administration, Supervision.

**Roberto Yukio Ikemoto**: Project administration, Writing - Review & Editing Formal analysis, Supervision.

## Research registration

N/a.

## Guarantor

Roberto Yukio Ikemoto.

## Provenance and peer review

Not commissioned, externally peer-reviewed.

## Declaration of competing interest

None of the authors has any conflict of interest to disclose.

We confirm that we have read the Journal's position on issues involved in ethical publication and affirm that this report is consistent with those guidelines.
